# Epithelial Sodium Channel-α Mediates the Protective Effect of the TNF-Derived TIP Peptide in Pneumolysin-Induced Endothelial Barrier Dysfunction

**DOI:** 10.3389/fimmu.2017.00842

**Published:** 2017-07-21

**Authors:** Istvan Czikora, Abdel A. Alli, Supriya Sridhar, Michael A. Matthay, Helena Pillich, Martina Hudel, Besim Berisha, Boris Gorshkov, Maritza J. Romero, Joyce Gonzales, Guangyu Wu, Yuqing Huo, Yunchao Su, Alexander D. Verin, David Fulton, Trinad Chakraborty, Douglas C. Eaton, Rudolf Lucas

**Affiliations:** ^1^Vascular Biology Center, Medical College of Georgia, Augusta University, Augusta, GA, United States; ^2^Department of Physiology and Functional Genomics, University of Florida College of Medicine, Gainesville, FL, United States; ^3^Division of Nephrology, Hypertension, and Renal Transplantation, Department of Medicine, University of Florida College of Medicine, Gainesville, FL, United States; ^4^Cardiovascular Research Institute, UCSF, San Francisco, CA, United States; ^5^Institute for Medical Microbiology, Justus-Liebig University, Giessen, Germany; ^6^Department of Pharmacology and Toxicology, Medical College of Georgia, Augusta University, Augusta, GA, United States; ^7^Department of Medicine, Medical College of Georgia, Augusta University, Augusta, GA, United States; ^8^Department of Physiology, Emory University School of Medicine, Atlanta, GA, United States

**Keywords:** epithelial sodium channel, non-selective cation channel, TNF, pneumonia, pneumolysin, endothelial barrier function

## Abstract

**Background:**

*Streptococcus pneumoniae* is a major etiologic agent of bacterial pneumonia. Autolysis and antibiotic-mediated lysis of pneumococci induce release of the pore-forming toxin, pneumolysin (PLY), their major virulence factor, which is a prominent cause of acute lung injury. PLY inhibits alveolar liquid clearance and severely compromises alveolar–capillary barrier function, leading to permeability edema associated with pneumonia. As a consequence, alveolar flooding occurs, which can precipitate lethal hypoxemia by impairing gas exchange. The α subunit of the epithelial sodium channel (ENaC) is crucial for promoting Na^+^ reabsorption across Na^+^-transporting epithelia. However, it is not known if human lung microvascular endothelial cells (HL-MVEC) also express ENaC-α and whether this subunit is involved in the regulation of their barrier function.

**Methods:**

The presence of α, β, and γ subunits of ENaC and protein phosphorylation status in HL-MVEC were assessed in western blotting. The role of ENaC-α in monolayer resistance of HL-MVEC was examined by depletion of this subunit by specific siRNA and by employing the TNF-derived TIP peptide, a specific activator that directly binds to ENaC-α.

**Results:**

HL-MVEC express all three subunits of ENaC, as well as acid-sensing ion channel 1a (ASIC1a), which has the capacity to form hybrid non-selective cation channels with ENaC-α. Both TIP peptide, which specifically binds to ENaC-α, and the specific ASIC1a activator MitTx significantly strengthened barrier function in PLY-treated HL-MVEC. ENaC-α depletion significantly increased sensitivity to PLY-induced hyperpermeability and in addition, blunted the protective effect of both the TIP peptide and MitTx, indicating an important role for ENaC-α and for hybrid NSC channels in barrier function of HL-MVEC. TIP peptide blunted PLY-induced phosphorylation of both calmodulin-dependent kinase II (CaMKII) and of its substrate, the actin-binding protein filamin A (FLN-A), requiring the expression of both ENaC-α and ASIC1a. Since non-phosphorylated FLN-A promotes ENaC channel open probability and blunts stress fiber formation, modulation of this activity represents an attractive target for the protective actions of ENaC-α in both barrier function and liquid clearance.

**Conclusion:**

Our results in cultured endothelial cells demonstrate a previously unrecognized role for ENaC-α in strengthening capillary barrier function that may apply to the human lung. Strategies aiming to activate endothelial NSC channels that contain ENaC-α should be further investigated as a novel approach to improve barrier function in the capillary endothelium during pneumonia.

## Introduction

Pulmonary permeability edema is a life-threatening complication of severe pneumonia and acute respiratory distress syndrome (ARDS), characterized by impaired alveolar liquid clearance (ALC) and alveolar–capillary hyperpermeability (1). Antibiotic treatment of patients infected with *Streptococcus pneumoniae* significantly reduces bacterial load, but it can also cause massive release of bacterial toxins in the lung compartment (2). The 53-kDa pneumococcal pore-forming virulence factor pneumolysin (PLY) was shown to be an important mediator of permeability edema, due to its capacity to impair both endothelial (3, 4) and epithelial barrier function (5). Although pneumococci release sufficient amounts of PLY to perforate the host cell plasma membrane, this does not necessarily cause immediate cell death, since membrane segments harboring toxin-induced pores can be either internalized or eliminated by microvesicle shedding. Dysregulation of cellular homeostasis secondary to transient pore formation/elimination is likely responsible for the damaging actions of PLY (6). To date, no proven treatment exists for increased pulmonary permeability edema, apart from ventilation strategies. Hence, the search for novel therapeutic agents that have the ability to restore both endothelial barrier function and ALC capacity is warranted.

Apart from impairing barrier function, PLY has also been shown to decrease the activity of the epithelial sodium channel (ENaC) (7), which is expressed on the apical side of alveolar epithelial cells and which, together with the basolaterally expressed Na^+^–K^+^-ATPase (8, 9), represents the primary mediator of Na^+^ uptake and liquid clearance in the alveolar compartment. In its native form, ENaC consists of three subunits, α, β, and γ (10, 11), but also a fourth δ subunit has been described, which can substitute for the α subunit (12). ENaC activity is defined as the product of its surface expression *N*, which is at least partially determined by Nedd-4-2-dependent ubiquitination (13) and its open probability *P*o, the latter of which is significantly increased by the formation of a complex comprised of ENaC subunits with MARCKS and PIP_2_ (14). In order to be fully functional, ENaC has to interact with the actin cytoskeleton and in particular with the actin-binding protein, filamin A (FLN-A) (15).

We recently demonstrated that the 17 residue circular TIP peptide (sequence: CGQRETPEGAEAKPWYC), which mimics the lectin-like domain of TNF, directly binds to two domains within the crucial α subunit of ENaC (16–18). The TIP peptide, through binding to residues Val567 and Glu568 increases the channel’s open probability time by promoting complex formation between human ENaC-α and MARCKS (18). In addition, the peptide augments ENaC-α surface expression in PLY-treated H441 cells, by means of reducing the subunit’s ubiquitination (18). This activity requires the presence of *N*-glycosylated Asn residues in the extracellular loop of the subunit (17). The presence of the TIP peptide has been shown to increase ALC and to ameliorate acute lung injury *in vivo* in several species (16, 19–23). The TIP peptide is well tolerated, and no significant side effects have been reported upon inhalation in healthy male volunteers (24). The TIP peptide is emerging as a potential therapeutic candidate for improving lung function. Data from two phase IIa clinical trials with inhalation of TIP peptide (a.k.a. AP301 and solnatide) in acute lung injury patients, the majority of which had severe pneumonia, and another trial in patients with primary graft dysfunction upon lung transplantation (www.ClinicalTrials.gov, Identifier NCT01627613 and NCT02095626, respectively) document efficacy. Both of these pathologies are characterized by capillary endothelial dysfunction.

Although originally thought to mainly constitute the rate-limiting entry step in Na^+^ reabsorption across lung, kidney, and colon epithelia, it has become clear in recent years that ENaC may also play an important role in the vasculature. In large vessels, ENaC is expressed in both endothelial and vascular smooth muscle cell compartments, where it operates as a mechano-sensitive channel, exposed to varying rates of blood flow and laminar shear stress (25). In contrast to large vessels, the presence or role of ENaC in the microvasculature, such as in the capillaries in the lung, remains understudied and represents the primary focus of this study. The TIP peptide was shown to increase Na^+^ uptake in pulmonary microvascular endothelial cells (26) and to restore impaired endothelial barrier function in the presence of the pore-forming toxins PLY and listeriolysin-O (3, 27).

In view of the previously observed protective activities of the TNF-derived TIP peptide on capillary barrier function in the presence of bacterial toxins, in this study, we investigated the role of its binding partner—ENaC-α—in microvascular endothelial cell barrier function. Our objective was to identify those common signaling molecules modified by bacterial toxins that are involved in both endothelial barrier impairment and ENaC dysfunction.

## Materials and Methods

### Cells

Human lung microvascular endothelial cells (HL-MVEC) were grown in complete EBM-2 medium (Lonza, Walkersville, MD, USA) at 37°C and 5% CO_2_. Experiments with PLY were performed in serum-free medium, since the toxin’s activity is neutralized by cholesterol.

### PLY Purification

Pneumolysin was purified from a recombinant *Listeria innocua* 6a strain expressing LPS-free PLY. The batch of PLY used in this study had a specific activity of 1.25 × 10^7^ hemolytic units per milligram.

### Biochemicals

Rabbit polyclonal anti-ENaC-α (59), β (60), and γ (2102) antibodies were generated in the laboratory of D.C.E (14), anti-human FLN-A, anti-human phospho-FLN-A, anti-human CaMKII, anti-human phospho-CaMKII, and anti-Actin HRP were from Cell Signaling Technology (Danvers, MA, USA). Rabbit anti-human ENaC-α was from Novus Biologicals (Littleton, CO, USA), a rabbit anti-human ASIC1 for IP was from EMD Millipore (Temecula, CA, USA), and a rabbit anti-hASIC1 antibody for WB was a kind gift from Dr. John Wemmie, University of Iowa. Goat anti-rabbit secondary antibodies conjugated to HRP were from Cell Signaling Technology (Danvers, MA, USA). MitTx was purchased from Alomone (Jerusalem, Israel), CaMKII inhibitor XII was from EMD Millipore (Billerica, MA, USA), and the TIP peptide was custom-ordered and purchased from AMBIOPHARM (North-Augusta, SC, USA).

### Depletion of ENaC-α or Acid-Sensing Ion Channel 1a (ASIC1a) in HL-MVEC

Human lung microvascular endothelial cells were treated with a pool of target-specific 19–25 nt siRNAs designed to knock down either ENaC-α conducting subunit or ASIC1a gene expression, and non-specific, non-targeting siRNA were obtained from Ambion (Grand Island, NY, USA). All siRNA’s were received in lyophilized form. HL-MVEC were transfected at 70–80% confluence with 50–75 nM final concentration of siRNA using siPORT™ Amine transfection reagent (Ambion, Life Technologies, Grand Island, NY, USA) and used for further experiments at 48 h post transfection.

### Immunoprecipitation

Human lung microvascular endothelial cells were grown in 60-mm culture flasks and were washed with PBS, scraped, and lysed in 400 µl of 20 mM Tris–HCl, pH 7.4 buffer containing 0.15 M NaCl, 1% non-idet P-40, 2 mM EDTA, as well as protease inhibitors. Lysates were incubated with empty beads in order to remove the non-specific binding partners (preclearing step) and subsequently with ASIC1 antibody for 1 h at 4°C. The mixture of the antibody and the precleared whole cell lysate was then incubated with agarose G magnetic beads overnight at 4°C, followed by three washing steps with PBS containing 2% BSA and eluted in 150 µl of Laemmli buffer. The resulting supernatants were analyzed by western blotting with ENaC-α antibody.

### Immunoblotting Procedure

Immediately after treatment, HL-MVEC were washed twice with ice-cold PBS and lysed with RIPA buffer containing a phosphatase and a protease inhibitor mixture. After centrifugation, clear supernatants were mixed with SDS sample buffer and boiled for 5 min. Protein extracts were separated on SDS/PAGE, transferred to a nitrocellulose membrane, incubated with primary antibodies, and subsequently after washing with HRP-conjugated secondary Ab. Immunoreactive proteins were visualized with Clarity solution (Bio-Rad, Hercules, CA, USA) and were then captured using ChemiDoc system (Bio-Rad). The relative intensity of each protein band was quantified using the ImageLab software (Bio-Rad).

### NanoPro Technology

Immediately after treatment, cells were washed and lysed with buffers from ProteinSimple (Santa Clara, CA, USA) as described previously (28). Preparation of cell lysates for size-based assay, using the Peggy system, was performed as described by the manufacturer (ProteinSimple).

### Measurement of Transendothelial Electrical Resistance

Transendothelial electrical resistance in HL-MVEC monolayers [electrical cell-substrate impedance sensing (ECIS) system 1600R; Applied Biophysics, Troy, NY, USA] was measured as described previously (3).

### Statistical Analysis

All experimental data are presented as mean ± SD. Control samples and those obtained upon various stimuli were compared by unpaired Student’s *t*-test. For multiple group comparisons, one-way ANOVA was used. Also, *p* < 0.05 was considered statistically significant.

## Results

### HL-MVEC Express All ENaC Subunits

**Figure 1:**
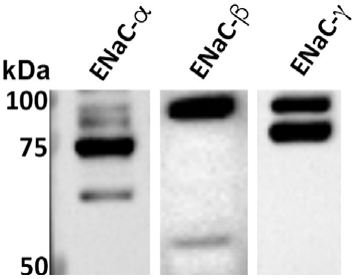
Representative western blot of basal expression of α, β, and γ epithelial sodium channel (ENaC) subunits in human lung microvascular endothelial cells. The immunoreactive bands at 95, 75, and 65 kDa are different forms of the subunits resulting from posttranslational modifications (e.g., glycosylation) and proteolytic processing. We previously confirmed the identity of similar bands excised from Coomassie stained gels after immunoprecipitation using the ENaC-α 59 and ENaC-β 60 antibodies and then performing LC/MS. We further corroborated the specificity of these antibodies by performing competition experiments using the recombinant fusion proteins that were used as the immunogens to generate the ENaC-α 59 and ENaC-β 60 antibodies (14). The characterization of the ENaC-γ 2102 antibody was described elsewhere (29). Although these ENaC antibodies cross-react with mouse, rat, and human species, we do expect there to be some variations in the previously reported sizes of the bands for ENaC expressed in the kidneys and, as shown here, in the lungs. These variations might be due to differences in proteases within the kidneys and lungs that cleave ENaC.

We previously demonstrated, using whole cell voltage-clamped patch clamp, that TIP peptide increased amiloride-sensitive Na^+^ currents in freshly isolated mouse MVEC (26). Here, we investigated whether HL-MVEC express the three ENaC subunits. Immunoblotting analysis revealed the presence of both uncleaved and mature ENaC-α, β, and γ subunits in human lung MVEC (Figure 1). The immunoreactive bands of ENaC-α at 95, 75, and 65 kDa represent different forms of the subunit, resulting from posttranslational modifications (e.g., glycosylation) and proteolytic processing.

### ENaC-α Expression Strengthens Barrier Function in PLY-Treated HL-MVEC Monolayers

**Figure 2:**
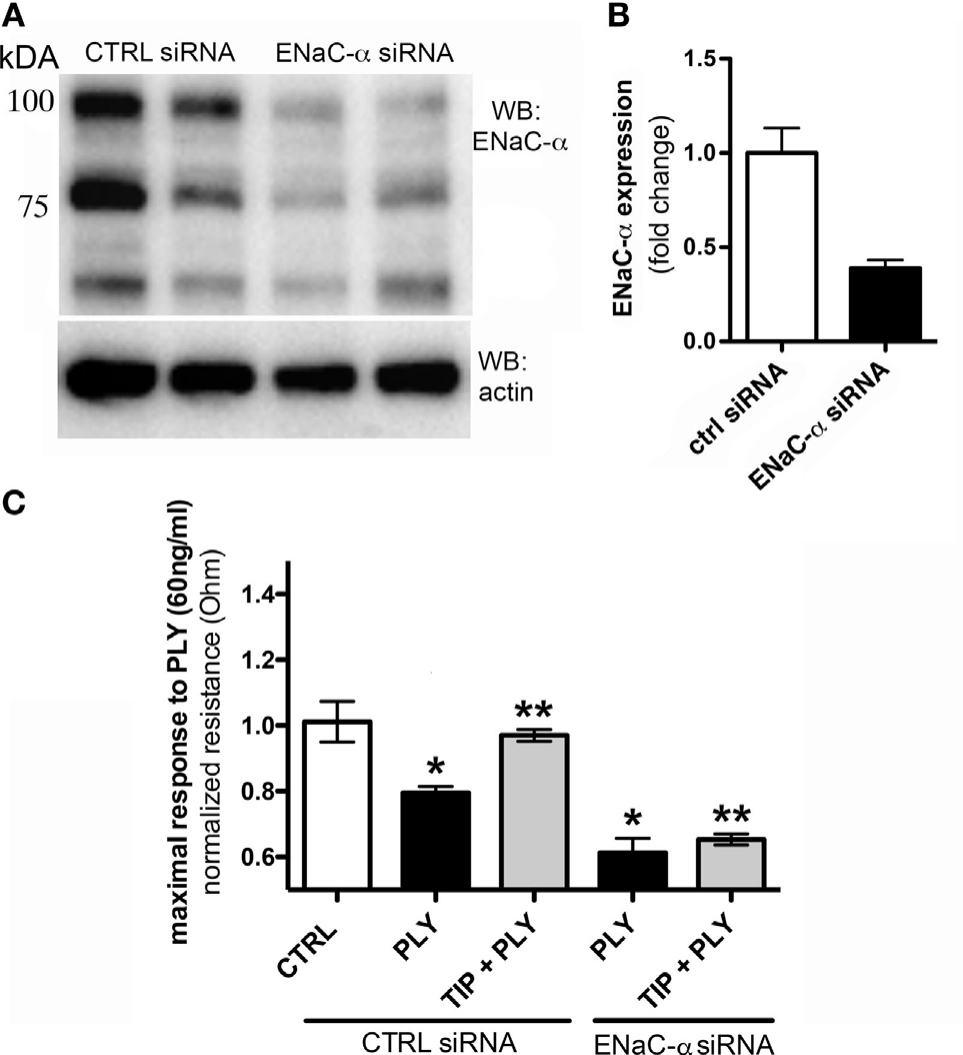
**(A)** Representative western blot of epithelial sodium channel (ENaC)-α expression in human lung microvascular endothelial cells (HL-MVEC), transfected with either scrambled siRNA (control) or ENaC-α siRNA. **(B)** Efficacy of siRNA-mediated depletion of ENaC-α, relative protein expression in HL-MVEC. **(C)** Transendothelial resistance (measured in electrical cell-substrate impedance sensing 1600R) in HL-MVEC, transfected with scrambled siRNA or ENaC-α siRNA and treated for 30 min with 60 ng/ml of PLY (corresponding with the maximal drop in resistance), in the presence or absence of TIP peptide (*p < 0.05 versus ctrl, **p < 0.05 versus PLY).

The association of ENaC with the cytoskeletal network at the apical membrane is required to help maintain its presence at this site and to prevent its removal by endocytosis (30, 31). It has not yet been investigated whether activation of the channel also affects endothelial barrier function. We therefore investigated whether ENaC expression in HL-MVEC monolayers affects barrier function, by depleting ENaC-α using specific siRNA, employing scrambled non-specific siRNA as a control. The efficacy of the siRNA-mediated depletion is shown in Figure 2A. As shown in Figure 2B, transfection with ENaC-α siRNA significantly reduced expression of ENaC-α in HL-MVEC, using the prominent 75 kDa band corresponding to the mature subunit for quantification. Depletion of ENaC-α significantly increased sensitivity of HL-MVEC to PLY at 30 min post addition of the toxin (Figure 2C). This was measured as normalized monolayer resistance, using ECIS (ECIS1600R, Applied Biophysics, Troy, NY, USA), in cells treated with 60 ng/ml PLY for 30 min. Silencing of ENaC-α did not affect basal barrier function (data not shown). The protective action of the TIP peptide (50 µg/ml) in PLY-induced barrier dysfunction, which we reported previously (3) and which was also observed in the presence of scrambled siRNA, was eliminated after depleting ENaC-α (Figure 2C). These results suggest an important role for ENaC-α in restoring capillary barrier function in the face of PLY challenge.

### ENaC-α Stimulation Blunts PLY-Induced CaMKII Activation and FLN-A Phosphorylation

Apart from its role in endothelial barrier function demonstrated above, ENaC-α has also been shown to be crucial for ALC (32), since mice lacking the subunit die shortly after birth with flooded lungs. ALC is, in part, modulated by ENaC activity. As such, we wanted to identify an interacting partner that binds to ENaC-α and that regulates both barrier function and Na^+^ uptake. The actin-binding protein FLN-A, in its non-phosphorylated form, is a prominent regulator of endothelial barrier function, since it prevents stress fiber formation (33). FLN-A directly associates with ENaC subunits and promotes their association with the chaperone protein MARCKS, thereby inducing complex formation of the channel with PIP_2_ (15). This complex formation is crucial for regulating the open probability of ENaC (14). Increased intracellular Ca^2+^ levels mobilize calmodulin, which in turn activates calmodulin-dependent kinase II (CaMKII), which then phosphorylates its substrate, FLN-A (15, 33). Phosphorylated FLN-A blunts the association of ENaC with MARCKS, and impairs ENaC activity (15).

**Figure 3:**
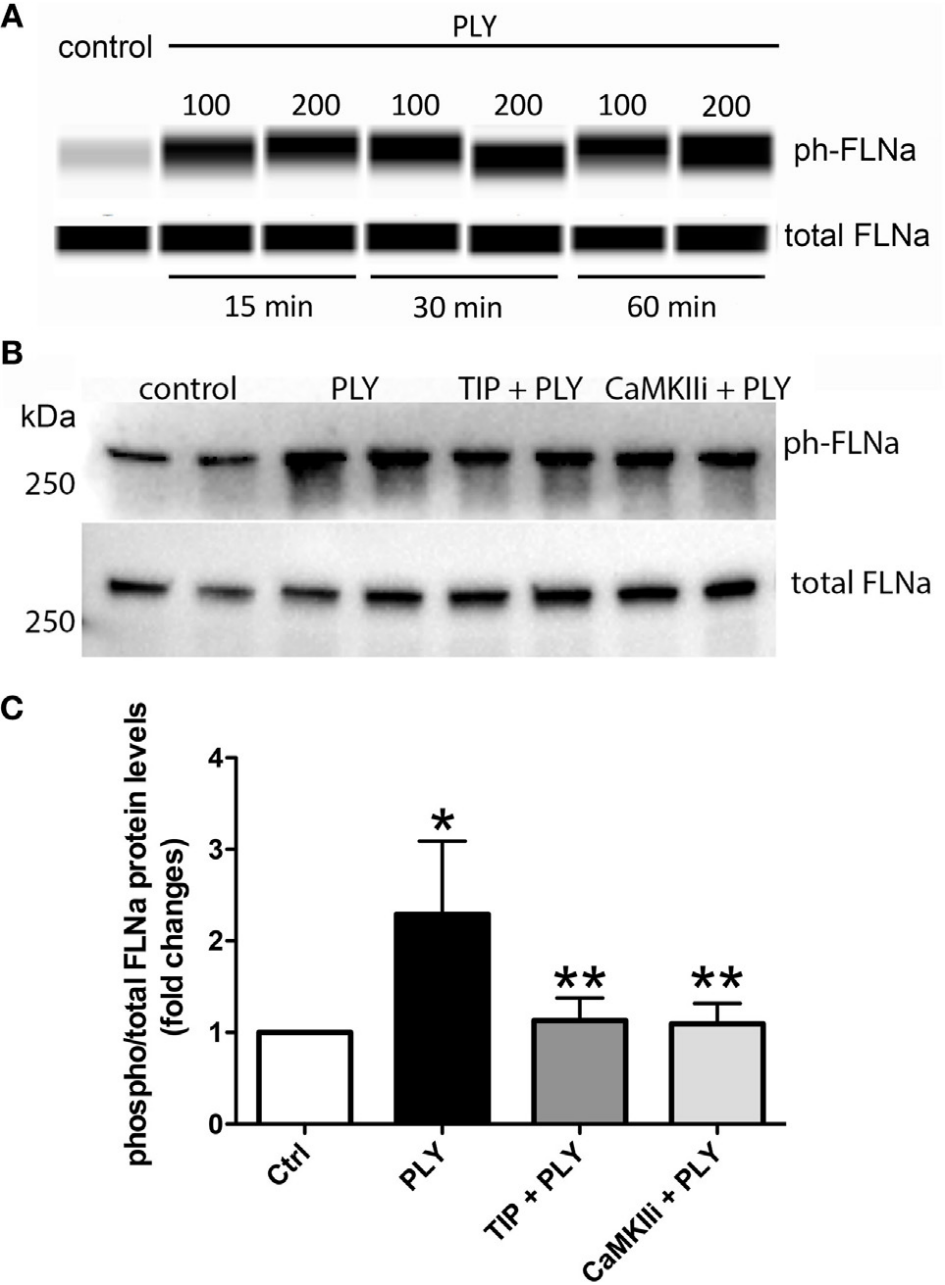
**(A)** Time-dependent representative nanopro technology-based western blot of PLY-induced filamin A (FLN-A) phosphorylation in human lung microvascular endothelial cells (HL-MVEC) (100 and 200 ng/ml), as described (28). **(B)** Representative western blot. **(C)** Quantification of phospho- over total protein ratio of PLY (60 ng/ml)-mediated FLN-A phosphorylation in HL-MVEC after 20 min. Cells were either pretreated with TIP peptide (50 µg/ml) or the CaMKII inhibitor (1 mM) for 15 min. Values are presented as means ± SD of three independent experiments in duplicates (*p < 0.05 versus ctrl, **p < 0.05 versus PLY).

**Figure 4:**
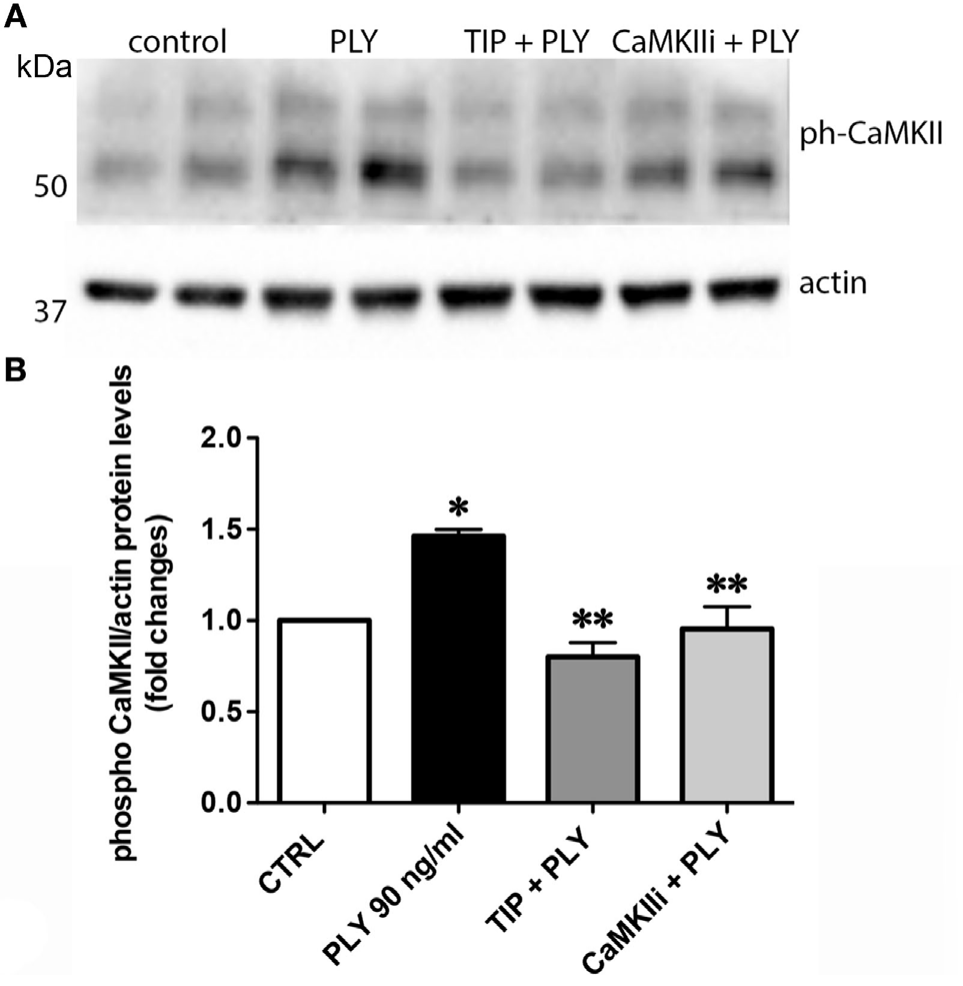
**(A)** Representative western blot and **(B)** quantification of phospho-CaMKII over actin ratio of PLY (90 ng/ml)-mediated CaMKII activation in human lung microvascular endothelial cells after 20 min. Cells were either pretreated with TIP peptide (50 µg/ml) or the CaMKII inhibitor XII (1 mM) for 15 min. Values are presented as means ± SD of three independent experiments in duplicates (*p < 0.05 versus ctrl, **p < 0.05 versus PLY).

Pneumolysin (100 or 200 ng/ml) induces FLN-A phosphorylation from as early as 15 min and persisting for at least 60 min (Figure 3A). TIP peptide (50 µg/ml), as well as the CaMKII inhibitor XII (1mM) inhibits FLN-A phosphorylation induced by PLY (60 ng/ml) (Figures 3B,C). As shown in Figure 4A, PLY-treatment (90 ng/ml) induces CaMKII activation in HL-MVEC within 10 min. Thus, both PLY-induced phosphorylation of FLN-A and CaMKII can be partially inhibited by the TIP peptide or by a CaMKII inhibitor (Figures 3A,B and 4A,B). Taken together, these data indicate that PLY, whose deleterious actions on barrier function in HL-MVEC monolayers are at least partially dependent on promoting Ca^2+^ influx (3), has the capacity to activate CaMKII, which in turn increases phosphorylation of FLN-A. TIP peptide binding to ENaC-α at least partially blunts these events.

### The Hybrid ENaC-α/ASIC1a Non-Selective Cation Channel Mediates Barrier Protection from PLY

In order to address the apparent discrepancy between our results with the TIP peptide, which improves barrier function in HL-MVEC, and results obtained by others demonstrating that aldosterone-induced activation of ENaC leads to stiffening in large vessel endothelial cells (34), we investigated the potential implication of other, non-selective cation channels (NSC) in the ability of the TIP peptide to preserve barrier function in PLY-treated HL-MVEC monolayers. Indeed, ENaC-α is not only a subunit of ENaC but also a component of hybrid NSC channels, where it forms a complex with the ASIC1a subunit (35–37). These hybrid NSC channels, when expressed in type 2 alveolar epithelial cells, were recently shown to contribute significantly to ALC (37).

**Figure 5:**
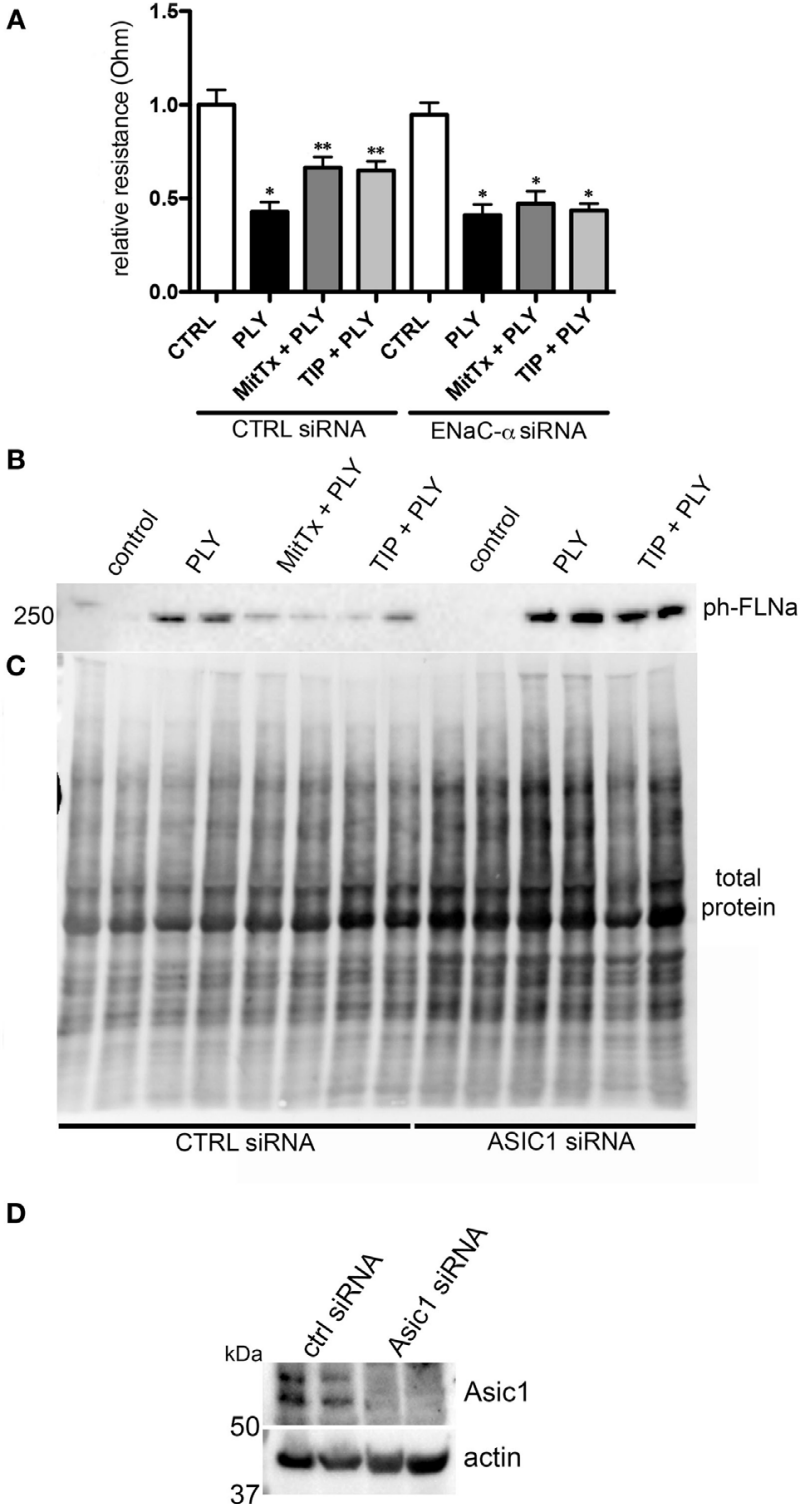
**(A)** Transendothelial resistance (measured in electrical cell-substrate impedance sensing 1600R) in human lung microvascular endothelial cells (HL-MVEC), transfected with scrambled siRNA or epithelial sodium channel (ENaC)-α siRNA and treated for 30 min with 60 ng/ml of PLY, in the presence or absence of TIP peptide (50 µg/ml) or MitTx (20 nM) (*n* = 3, SEM) (*p < 0.05 versus ctrl, **p < 0.05 versus PLY). **(B)** Representative western blot and quantification of phospho- over total protein ratio of PLY (60 ng/ml)-mediated filamin A (FLN-A) phosphorylation in HL-MVEC after 20 min. Cells were either pretreated with TIP peptide (50 µg/ml) or MitTx (20 nM) for 15 min. **(C)** Representative stain-free blot showing total protein transferred to the nitrocellulose membrane. **(D)** siRNA-mediated acid-sensing ion channel 1a (ASIC1a) silencing in HL-MVEC.

**Figure 6:**
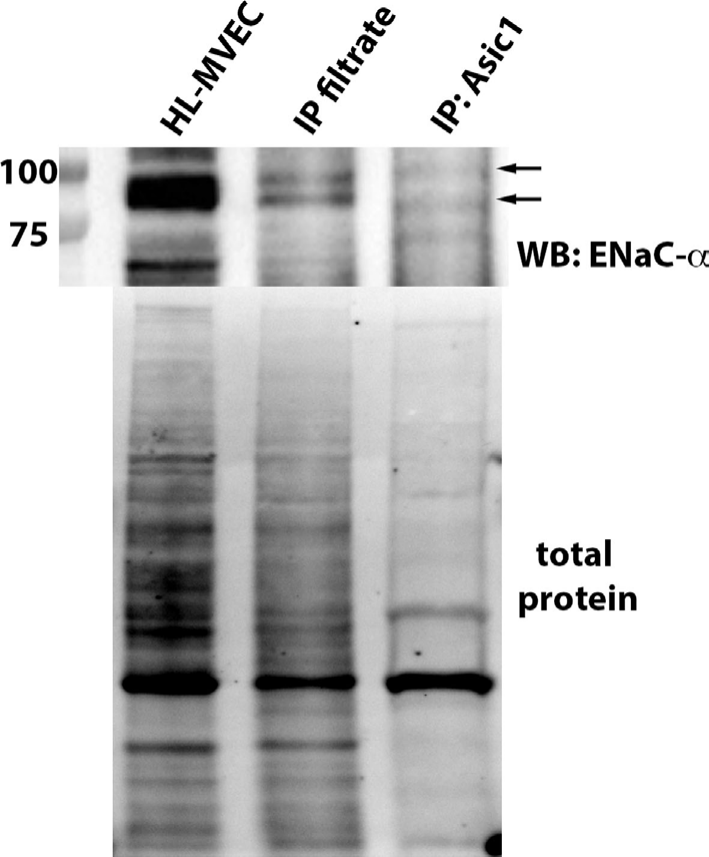
Representative immunoprecipitation experiment assessing binding of epithelial sodium channel (ENaC)-α (indicated by arrows) to native ASIC1 in human lung microvascular endothelial cells (HL-MVEC) and representative stain-free blot showing the total protein transferred to the nitrocellulose membrane before the co-IP experiment (whole HL-MVEC lysate—first lane), the filtrate after the co-IP (second lane), and the eluent (eluted from the ASIC1 antibody-decorated magnetic beads—third lane).

MitTx (20 nM), an activator of ASIC1a and of NSC (38, 39), significantly reduced PLY-mediated (60 ng/ml) barrier dysfunction in HL-MVEC, to the same extent as the TIP peptide (50 µg/ml) (Figure 5A). siRNA-mediated depletion of ENaC-α abrogated the protective effect of both MitTx and TIP peptide in PLY-treated HL-MVEC monolayers (Figure 5A). Moreover, siRNA-mediated depletion of ASIC1a, which is expressed in HL-MVEC (Figure 5D) abrogated the inhibitory effect of the TIP peptide on PLY-induced FLN-A phosphorylation (Figures 5B,C). Of note, PLY induced significantly higher FLN-A phosphorylation in cells lacking ASIC1a (Figure 5B). These results indicate that NSC channels at least partially mediate barrier protection against PLY in HL-MVEC and that both ENaC-α and ASIC1a subunits are crucial for this activity. To confirm this interaction, we performed a co-IP experiment using ASIC1a antibody as bait. We detected two bands corresponding to uncleaved (95 kDa) and mature (around 75 kDa) ENaC-α in the immunoprecipates (Figure 6).

## Discussion

Decreased lung capillary barrier function represents one of the major complications of severe pneumonia and ARDS and promotes the development of permeability edema. Upon autolysis or antibiotic-induced lysis, the G^+^ pathogen *S. pneumoniae*, the main etiological agent of community acquired pneumonia in the US, releases the cholesterol-binding and pore-forming toxin PLY.

Pneumolysin-induced Ca^2+^-influx, which is blunted by lanthanum chloride, is crucial for the ability of the toxin to induce hyperpermeability in human lung MVEC (3). PLY reduces endothelial barrier function in part by means of activating protein kinase C-α, which in turn impairs NO generation by endothelial nitric oxide synthase (eNOS) (3, 4), which was shown to be required for basal barrier function (40). Increased Ca^2+^ influx can also mobilize calmodulin, which activates CaMKII. Activated CaMKII phosphorylates the actin-binding protein FLN-A. Although the non-phosphorylated form of FLN-A prevents stress fiber formation, increased Ca^2+^ influx promotes the shift to its phosphorylated form, which is incapable of supporting barrier integrity (15).

**Figure 7:**
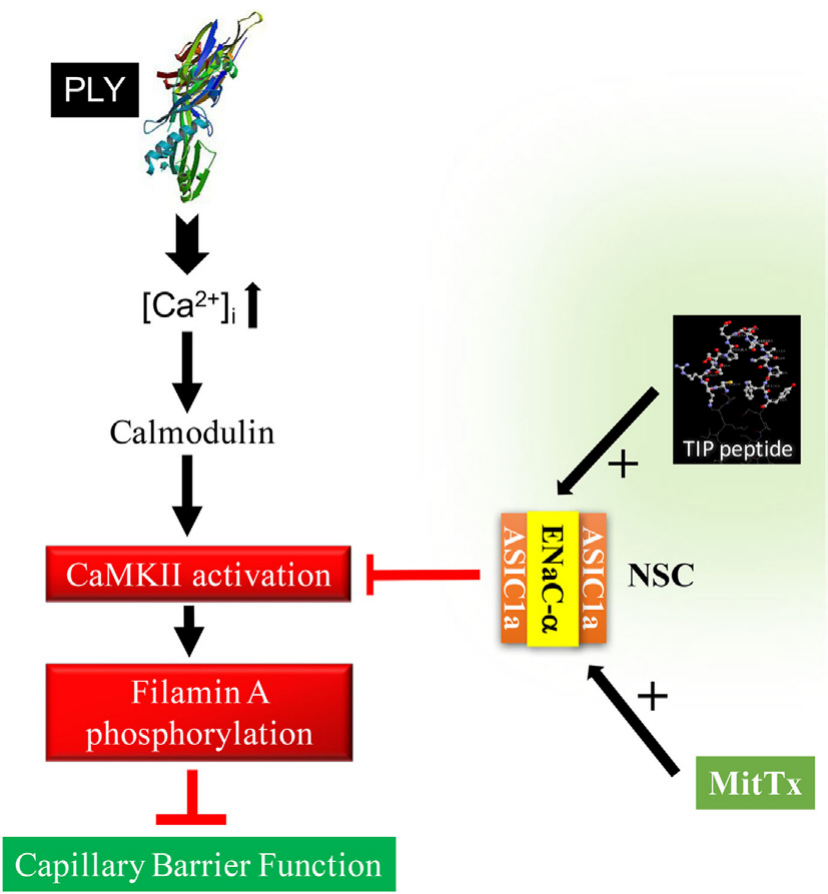
Proposed sequence of events in the role of epithelial sodium channel (ENaC)-α in barrier protection in pneumolysin (PLY)-treated human lung microvascular endothelial cells. PLY, upon pore formation, increases Ca^2+^-influx (3), which in turn mobilizes calmodulin. Calmodulin activates CaMKII, which in turn phosphorylates its substrate filamin A (FLN-A) (15). Phosphorylated FLN-A promotes stress fiber formation and increases capillary permeability. Activation of NSC, by either TIP peptide (binding to ENaC-α) or MitTx [binding to acid-sensing ion channel 1a (ASIC1a)], abrogates PLY-mediated CaMKII activation and protects as such from PLY-induced hyperpermeability.

Apart from preventing stress fiber formation, FLN-A also promotes the interaction between the chaperone protein MARCKS and ENaC subunits (15), which in turn increases the open probability time of the channel. As summarized in Figure 7, our findings suggest that ENaC-α, as a subunit of NSC, can be activated by the TIP peptide, whereas the ASIC1a subunit of NSC is activated by MitTx. Both of these mechanisms of NSC activation promote barrier protection, by means of reducing PLY-induced activation of CaMKII, FLN-A phosphorylation, and finally lung capillary barrier dysfunction, respectively. A role for ENaC-α in epidermal barrier protection was shown previously (41). Of note, a recent study demonstrated that both the β1 subunit of the Na^+^–K^+^-ATPase as well as the α subunit of ENaC strengthen capillary endothelial barrier function in mice in the presence of LPS (42). These data together with those presented here suggest an important role for ENaC-α in protecting endothelial barrier function in the presence of bacterial toxins.

Our results with the TNF-derived TIP peptide, which directly binds to ENaC-α and which has the capacity to increase both expression and open probability of ENaC in the presence of PLY, are in sharp contrast to the suggested role of ENaC in aldosterone-induced vascular stiffening and eNOS dysfunction in large vessel endothelial cells (34). Although it cannot be excluded that aldosterone, apart from activating ENaC also activates other pathways possible leading to endothelial dysfunction (43), and that large vessel endothelial cells, as investigated in the Kusche-Vihrog studies, are phenotypically different from microvascular endothelial cells, our results indicate that ENaC-α participates in barrier strengthening in HL-MVEC at least partially in the context of a complex different from classical ENaC.

Acid-sensing ion channels represent a family of proteins activated upon extracellular acidification (35). Although primarily found in neurons, ASIC1 expression was also demonstrated in type 2 alveolar epithelial cells, where they play an important role in vectorial Na^+^ transport-mediated ALC (37), as well as in cerebral arteries (44) and in pulmonary arterial smooth muscle cells (45). We demonstrate here that human pulmonary microvascular endothelial cells also express ASIC1a. MitTx, an ASIC1 and NSC activator, which does not interact with ENaC, strengthens barrier function in PLY-treated HL-MVEC. However, the protective effects of MitTx are blunted in cells in which ENaC-α was depleted, indicating that its actions are not mediated by the typical ASIC1a channel complex, but rather by an ASIC1a/ENaC-α hybrid. Moreover, the inhibitory effect of the TIP peptide on PLY-induced FLN-A phosphorylation is abrogated in cells in which ASIC1a was depleted, indicating that the classical ENaC channel complex is not sufficient to mediate its effect. These results indicate that, rather than the classical ASIC1a and ENaC channels, a hybrid NSC channel, consisting of both ASIC1a and ENaC-α, is likely to mainly mediate the protective effects of both the TIP peptide and MitTx in lung capillary endothelial cells. This mechanism could be especially relevant in conditions of acidification, as can be found during bacterial pneumonia. Acidification of exhaled breath condensate in ventilated acute lung injury and ARDS patients was shown to correlate with local pulmonary inflammation (46). As such, it seems plausible that under these conditions, ASCIC1a, as well as NSC can be activated.

In conclusion, our data indicate that the barrier protective effect of ENaC-α in PLY-treated HL-MVEC monolayers is at least partially mediated by NSC channels in these cells. As such, the TIP peptide, which has the capacity to activate both ALC across alveolar epithelium and endothelial barrier function in the presence of bacterial toxins, could represent a therapeutically promising candidate to tackle pulmonary permeability edema associated with bacterial pneumonia. *In vivo* studies will be needed to further test this mechanism under pathologically relevant conditions.

## Author Contributions

Conception or design of the work: IC, AAA, MM, DF, TC, DE, and RL; acquisition, analysis, or interpretation of data: IC, SS, BG, HP, MH, BB, MR, and JG: drafting the work: IC and RL; revising it critically for important intellectual content: AAA, MR, GW, YH, YS, AV, DF, and DE. All authors approved the final version of the manuscript and agreed to be accountable for all aspects of the work in ensuring that questions related to the accuracy or integrity of any part of the work are appropriately investigated and resolved.

## Conflict of Interest Statement

No conflicts of interest, financial or otherwise, are declared by the authors. The reviewer, SH, declared a past coauthorship with one of the authors, TC, to the handling Editor, who ensured that the process met the standards of a fair and objective review.
